# Reorganization of the Connectivity of Cortical Field DZ in Congenitally Deaf Cat

**DOI:** 10.1371/journal.pone.0060093

**Published:** 2013-04-12

**Authors:** Pascal Barone, Ludovic Lacassagne, Andrej Kral

**Affiliations:** 1 Université Toulouse, CerCo, Université Paul Sabatier, Toulouse, France; 2 CNRS, UMR 5549, Toulouse, France; 3 Laboratory of Auditory Neuroscience, Department of Experimental Otology, Institute of Audioneurotechnology, Medical University Hannover, Hannover, Germany; 4 Laboratory of Auditory Neuroscience, Institute of Neurophysiology, University Clinics Hamburg-Eppendorf, Hamburg, Germany; University of Salamanca- Institute for Neuroscience of Castille and Leon and Medical School, Spain

## Abstract

Psychophysics and brain imaging studies in deaf patients have revealed a functional crossmodal reorganization that affects the remaining sensory modalities. Similarly, the congenital deaf cat (CDC) shows supra-normal visual skills that are supported by specific auditory fields (DZ-dorsal zone and P-posterior auditory cortex) but not the primary auditory cortex (A1). To assess the functional reorganization observed in deafness we analyzed the connectivity pattern of the auditory cortex by means of injections of anatomical tracers in DZ and A1 in both congenital deaf and normally hearing cats. A quantitative analysis of the distribution of the projecting neurons revealed the presence of non-auditory inputs to both A1 and DZ of the CDC which were not observed in the hearing cats. Firstly, some visual (areas 19/20) and somatosensory (SIV) areas were projecting toward DZ of the CDC but not in the control. Secondly, A1 of the deaf cat received a weak projection from the visual lateral posterior nuclei (LP). Most of these abnormal projections to A1 and DZ represent only a small fraction of the normal inputs to these areas. In addition, most of the afferents to DZ and A1 appeared normal in terms of areal specificity and strength of projection, with preserved but smeared nucleotopic gradient of A1 in CDCs. In conclusion, while the abnormal projections revealed in the CDC can participate in the crossmodal compensatory mechanisms, the observation of a limited reorganization of the connectivity pattern of the CDC implies that functional reorganization in congenital deafness is further supported also by normal cortico-cortical connectivity.

## Introduction

Psychophysical and neuroimaging studies in both animal and human subjects have demonstrated that sensory deprivation from early developmental stages leads to a functional reorganization of the brain favoring the spared modalities [1]. Such crossmodal reorganization after sensory loss is based on plastic properties of the brain that allow adaptation to changes in the sensory environment (reviewed [2], [3]). The behavioral consequence of the crossmodal compensation is an enhancement of the perceptual skills in the remaining modalities. In early deaf subjects numerous psychophysical studies revealed visual abilities that surpass those normally reported in hearing subjects [4], while such improvements were rather limited to an enhanced "reactivity" to visual events [5] and might more specifically concern attention [6]. Such crossmodal compensation of perception is accompanied by functional reorganizations [7] expressed as a colonization of the deprived cortical areas by the remaining modalities. Brain imaging studies in deaf individuals have revealed that some deprived auditory areas can be activated by visual speech information such as sign language and lipreading [8]–[10] or even simple visual moving stimuli [11].

Animal models of sensory loss provide similar observations to those described in deaf or blind human subjects. Recently, area-specific visual compensations have been demonstrated in an animal model of congenital deafness – the congenitally deaf cat [12]. The congenitally deaf cat (CDC) suffers from a degeneration of the inner ear at birth while the auditory nerve is preserved, making these animals a good model to study cortical plasticity induced by congenital deafness and chronic stimulation with cochlear implants [13], [14]. Using a large battery of behavioral visual paradigms, it has been demonstrated that the CDCs have acquired specific visual compensation: firstly, the deaf cats show visual localization which exceeds hearing cats in the most peripheral visual field (60–90° eccentricity; [12]). Secondly, in the CDCs, the threshold for movement detection of a visual target is significantly lower than that reported in control cats (ibid.). Further, using a cooling device to inactivate restricted cortical areas, the study revealed that this visual compensation is supported by “auditory“ fields DZ (dorsal zone) and P (posterior auditory cortex). Altogether, this work, in agreement with human studies [15], [16], attests that crossmodal compensation following blindness or deafness is based on the recruitment of the cortical areas deprived of sensory inputs. In the deaf cats the visual compensation can thus be assigned to specific auditory areas [12].

The neuronal mechanism underlying such crossmodal cortical reorganization is still poorly understood. A common hypothesis postulates that crossmodal compensation following congenital sensory loss relies on the reorganization of the brain connectivity during the early stages of cortical maturation [17]. In support to this is the observation of altered cortical and thalamic connectivity of the brain of experimental animal models of blindness [18]–[20]. In case of congenital deafness, the knowledge of the anatomical substrate responsible for crossmodal compensation is limited [21], [22], while recent work in human suggests that it can involve different stages of visual processing down to the retina [23].

Two assumptions can be proposed that would account for crossmodal reorganization after congenital deafness. Crossmodal compensation might results from a reorganization of the connectivity of the auditory areas at either cortical or subcortical levels. Alternatively, functional reorganization could rely on changes in synaptic efficiency of normal, existing connections such as the heteromodal connections recently described. In order to answer this question, we performed an anatomical study to analyze the changes in connectivity pattern of the deafferented auditory cortex of adult congenitally deaf cats [12]. A histological analysis was performed by means of injections of anatomical tracers in adult CDC. Based on the previous behavioral study in deaf cats [12] we targeted the area DZ, known to be cross-modally reorganized in CDCs [12]. While similarly to area DZ, the posterior auditory cortex P is involved in crossmodal compensation, we did not perform dye injections into this area. This was motivated by the strategy of analyzing the entire connectivity targeting DZ and A1 including the projection arising from field P which would have been not available after receiving such an injection.

The involvement of the primary auditory area A1 in visual crossmodal reorganization remains controversial in humans and is probably negligible. In deaf patients some studies reported activation in secondary auditory areas but not in A1 during visual sign language or speechreading [10], [24] while other studies found that at least part of A1 can be activated by simple moving visual stimuli [11]. In the deaf cat, single unit recording in A1 failed to reveal visual responses [25], [26] while it was previously observed using ERPs recording [27]. Further, when the primary auditory cortex A1 was transiently inactivated, it did not affect the visual performances of the CDC [12]. In contrast, after the cooling of auditory area DZ, the performances of the deaf cats in movement detection dropped off to the level observed in hearing cat. In consequence, based on these functional considerations observed in the deaf cat, at the connectivity level we might expect that A1 and DZ will be differently affected by the congenital deafness.

## Methods

### Ethics Statement

All experimental protocols, including care and surgery of animals, were performed in strict accordance with the recommendations of the German state authorities on the use of laboratory animals. The protocol was approved by the Committee on the Ethics of Animal Experiments of the University of Hamburg (Office of Health and Consumer Protection of the State of Hamburg, permit number #23/06) and complied with guidelines of the European Ethics Committee on Use and Care of Animals.

### Surgical procedures and injections of retrograde tracers

Normal-hearing cats (NHC, controls) and congenitally deaf cats (CDC) were obtained from the breeding colony of the University Clinics Hamburg-Eppendorf. The present study is based on 16 injections of retrograde dyes performed in four adult cats, 2 normal and 2 congenitally deaf cats. All animals were adult (more than one year old). The deafness of the CDCs was established during a screenings at the age of 4 weeks by the absence of auditory-evoked brainstem responses (ABRs) to clicks and tone-pips of intensities up to 125 dB SPL. These deaf animals show a Scheibe-type of cochlear dysplasia, including complete loss of inner and outer hair cells, displacement of the tectorial membrane, collapse of the Reissner's membrane, but a good preservation of the spiral gangion cells (reviewed in [24]).

Prior to surgery, the animals were preanaesthetized with ketamine (24.5 mg/kg), xylazine (2.1 mg/kg) and atropine (0.2 mg/kg) i.m. Dexamethasone (0.3 mg/kg) was also administrated to prevent cerebral oedema. During surgery, heart rate, respiration rate and body temperature were monitored, the latter being maintained at 38°C using an electronically-controlled heating blanket. The anesthesia level was continuously checked based on physiological parameters (respiration rate, ECG, heart rate and capnometry, including acoustic alarms if the parameters exceeded the physiological range) and the presence of an areflexic state (checked in 15–20 minutes intervals). If necessary, additional doses of ketamine hydrochloride (12 mg/kg) were administrated. The head of the animal was then fixed in a holder. The skin was cut, the temporal muscle retracted, and a craniotomy performed above the auditory cortex and the dura mater was opened. Anatomic landmarks, in particular the suprasylvian sulcus (SSS), the anterior and posterior ectosylvian sulcus (AES and PES) were used to guide the injections of tracers in the different areas of interest. Because of the variability in the cerebral cat sulci of the cat [28], the success in injecting correctly the dye in A1 or DZ was verified by analyzing the pattern of retrogradely labeled cells in the thalamic nuclei with respect to the published data [29]. During surgery, the brain was photographed for documentation of the injections positions.

After completion of injections an artificial dura was positioned over the exposed cortex, the bone of the skull was put back and then covered by dental acrylic. The temporalis muscles and the skin were sutured. Each animal was monitored closely during 10–11 days survival time corresponding to the optimal period for the transport of the tracers. The animals recovered within 48 hours and, if required, received subcutaneous injections of fluids (physiological NaCl solution).

**Figure 1:**
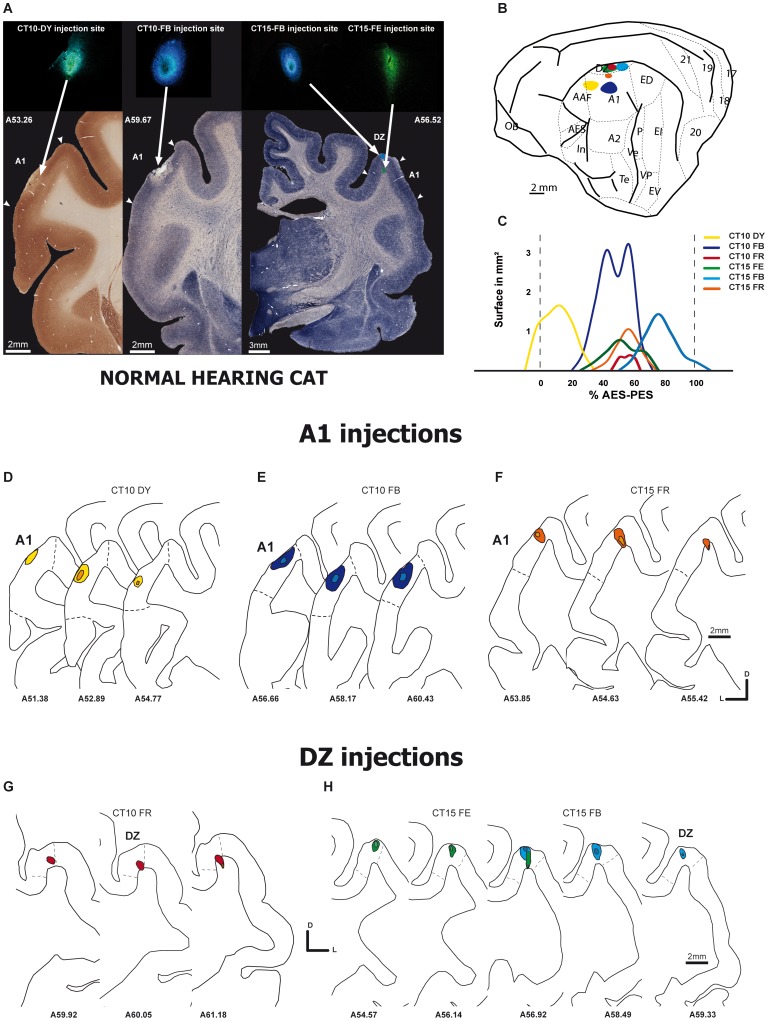
Injection sites in Normal hearing cats. **A**. Photomicrographs of frontal sections showing the location of three injections sites in A1 (left and middle panels) and DZ (right panel). The sections were processed for cytochrome oxidase (left panel) or Alkaline phosphatase. **B**. Schematic view of a cat brain areas. Each blob indicates a single injection located in A1 or DZ. In **C** are represented the 2D reconstructions of the individual dye injections according to their location (normalized distances) with respect to the distance that separate the anterior and posterior ectosylvian sulci (AES and PES). The color lines represent the antero-posterio extent of the pick-up zone of the retrograde dyes, and each of them (3 injections in case CT10 and 3 injections in case CT15) are represented with a different color. The lower graphs **D**–**H** represent the reconstruction on serial sections of the injection sites in A1 (D–F) and DZ (G–H). In each panel the case number are indicated (se table 1). The number of individual sections are indicated and the low to high numbers underneath the sections, represent the antero-posterior location of individual sections.

**Figure 2:**
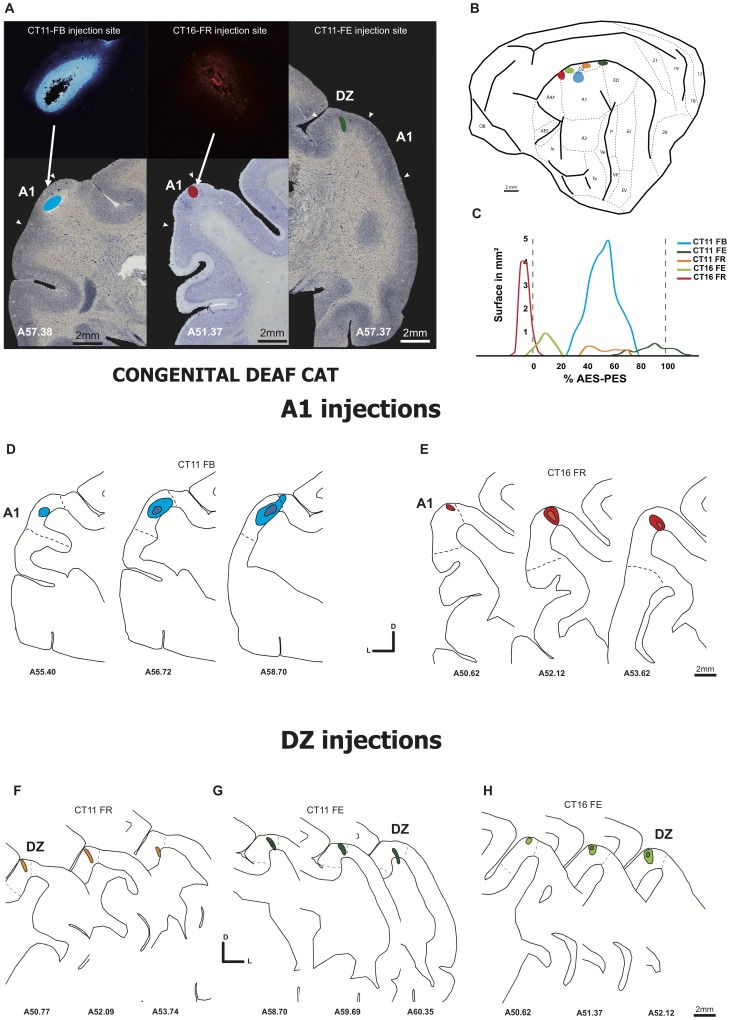
Injection sites in congenital deaf cats. **A**. Photomicrographs of frontal sections reacted for Alkaline phosphatase showing three injections in A1 (left and middle panels) and DZ (right panel). **B**. Schematic illustration of cat brain areas. Each injection site in A1 and DZ is indicted by a colored blob. **C.** 2D reconstructions of the individual 5 dye injections (3 in case CT11 and 2 in case CT16). The lower panels **D**–**H** represent the reconstruction on serial sections of the injection sites in A1 (D–E) and DZ (F–H). Convention as in figure 1.

**Figure 3:**
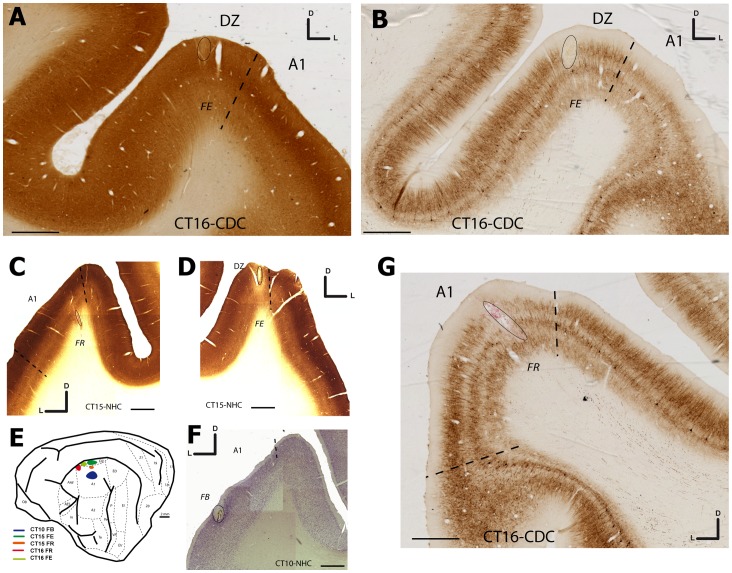
Microphotographies showing the location of the injection sites in A1 and DZ. In A and B is shown the FE injection in DZ of the deaf cat (CT–16) with respect to the A1/DZ border revealed by cytochrome oxidase (A) and SMI-32 (B). In G is shown the location of the FR injection in A1 on a cortical section reacted for SMI-32. The A1/DZ border is assessed by the presence of a rich neuropile staining in the upper layers of DZ as well as the presence of large reactive cells in layer V [35]. In panels C, D and F are illustrated the location of the injection sites in the NHC in A1 (C and F) or DZ (D) after Nissl (F) or CO staining (C–D). E shows a schematic view of a cat brain in which each blob indicates a single injection illustrated in this figure. Scale bars: 2 mm.

The animals received, in both hemispheres, injections of different retrograde tracers (see table 1 for details and Fig 1–3 for illustration of individual injections). In each animal, we used 4 different types of retrograde dyes with similar sensitivities and characteristics [30], [31]: fast blue (FB), di-amino yellow (DY), fluororuby (FR) and fluoroemerald (FE). Hamilton syringes, in some cases equipped with a glass micropipette (50–90 µm inner diameter) were used to inject 0.3–1 µl of retrograde fluorescent tracers: fast blue (FB-3% in NaCl), di-amino yellow (DY-3% in NaCl), fluororuby (FR-10% in H_2_O) and fluoroemerald (FE-10% in H_2_O). Using a syringe holder attached to the head holder frame, the injections were made perpendicularly to the cortical surface at a depth of about 600–800 µm and consisted of single or multiple injections of dyes. In case of multiple injections, they were performed during a single penetration but at different cortical depth. Injections were performed ventrally at a distance of 1–3 mm to the SSS, caudally or rostrally at the level of the PES or AES. After the histological procedure, we localized the different injection sites in a region that identified as the dorsal region of the auditory cortex. This region is limited dorsally by the SSS, at 1–2 mm in front of the AES and caudally at the level of PES (figure 1–3).

**Table 1:** Description of the injection sites. FB: Fast Blue; DY: di-amino yellow, FE: fluoroemerald, FR: fluororuby.

Case	Hemisphere	Tracer (µl)	Area
**CT10-NHC**	Left	FB simple (0.3)	A1
	Left	DY simple (0.4)	A1
	Right	FR simple (0.8)	DZ
**CT15-NHC**	Left	FR simple (0.6)	A1
	Right	FB simple (0.4)	DZ
	Right	FE multiple (0.9)	DZ
**CT11-CDC**	Left	FB simple (0.5)	A1
	Right	FE simple (0.5)	DZ
	Right	FR simple (0.5)	DZ
**CT16-CDC**	Left	FR multiple (0.8)	A1
	Left	FE multiple (0.9)	DZ

The cerebral cortex of the cat [32] presents a large variability of location of the cortical regions with respect to the surface landmark (gyri and sulci). In our case we have placed our injection in areas A1 and DZ with respect to the SSS, AES and PES sulci. In our 4 experimental animals the dorsal AES-PES distance is of about 6.4±0.73 mm in agreement with previous observation [28]. Therefore, we are providing the location of the injection sites with respect to a normalized distance separating the AES and PAS succi (see Fig 1C and 2C).

### Histological processing

After the survival period (10–11 days), animals were given a lethal dose of pentobarbital before being perfused intracardially with 0.9% saline containing 0.1% heparin, followed by 4% paraformaldehyde in phosphate buffer pH 7.4 (PB), and with PB sucrose solutions of increasing concentrations (10, 20 and 30%) for cryoprotection. Brains were immediately removed and put in a PB solution of 30% sucrose over night and until the histological processing. Frontal serial sections (40 µm thick) were made of freezing microtome. Alternate sections were reacted for neuronal alkaline phosphatase (NAP, [33], cytochrome oxydase (CO; [34], SMI-32 [35], [36] or stained with Cresyl Violet.

### Data analysis

Sections were analyzed using light or fluorescent microscopy with a Leica microscope (DMR) equipped with a CCD camera. Each fluorescent dye presents a unique fluorochrome that under a specific excitation wavelength results in a specific emission wavelength that can be visualized with a specific filter. The characteristics of observation for each dye are: FB emission: 420nm, filter A Leica; DY emission: 390 nm, filter D Leica; FR emission: 580nm, filter L5 Leica; FE emission: 517 nm, filter N3 Leica.

Labeled neurons were counted based on the identification of a nucleus. For each cortical area the exact position of labeled neurons was computed on individual sections observed at regular intervals using Mercator software (Explora Nova). Sampling frequencies of analyzed sections were adjusted to the size of the areas containing labeled neurons (defined as the projection zone [37]). This allowed constructing two types of graphical representation of the data. First, we assessed the proportion (in %) of the number of labeled neurons in a given area with respect to the total of projecting cells observed in all areas projecting to the injection site. These values (% of fraction labeled neurons, [38]) are independent of the size of the injection site. Second, we constructed a density profile for the individual projections, representing the distribution of the number of retrogradely labeled neurons counted in individual sections across a cortical area. Area and laminar location of projection neurons was performed using the different adjacent sections stained with histological markers. The position of each section in the same animal was estimated using scale photography of the hemisphere and expressed with distance percentage.

**Figure 4:**
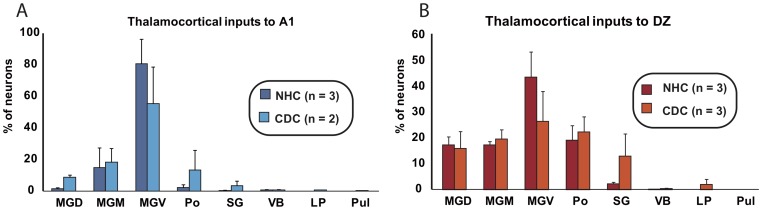
Histograms of the means of the distribution (in % of total, ± se) of projecting cells in the thalamic nuclei following an injection in A1 (A) or DZ (B). Values for normal (NHC) and deaf (CDC) cats are presented separately. No striking differences are observed in the proportions beside an abnormal projection in the deaf cats from the Lateral Posterior nucleus (LP) to A1. The number of injections sites in each group is indicated (n).

**Figure 5:**
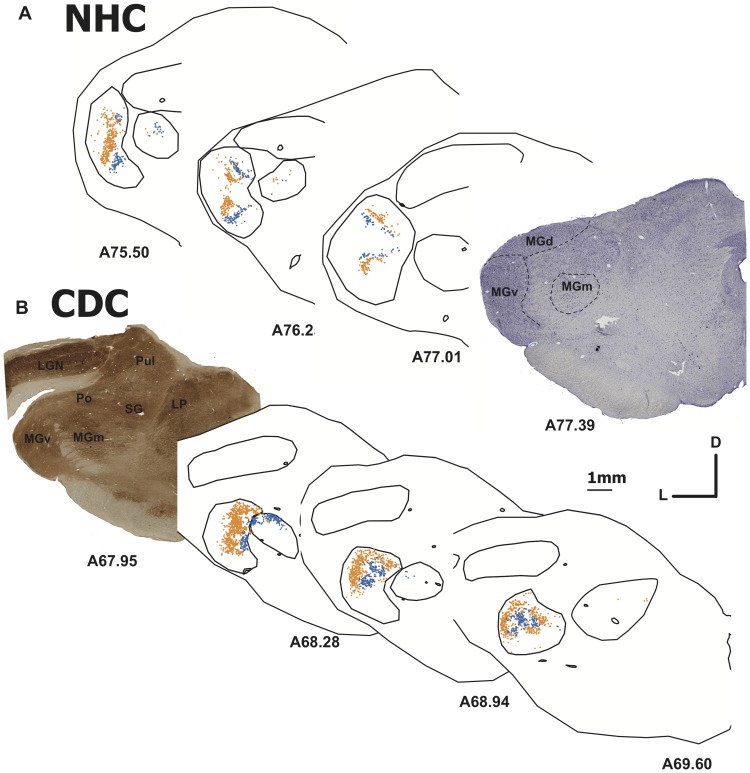
Tonotopic organization of the MGB projections to A1 in the normal (A, case CT10) and deaf (B, CT11) cat. Following a double injection of FB and DY in A1 the distribution of labeled cells are segregated across the MGB in both the normal and deaf cat. In each panel a single dots represents the location of a retrogradely labeled cell following a FB (blue) or a DY (yellow) injection. Nissl (A) and cytochrome oxydase (B) histological staining are shown to identify the location of the thalamic nuclei. Abbreviations: D dorsal, L lateral.

**Figure 6:**
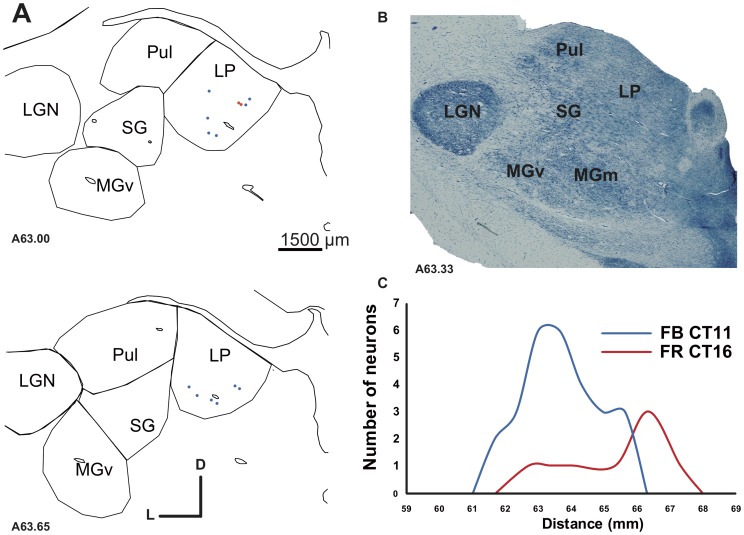
Projection from the Lateral Posterior nucleus (LP) in the deaf cat. In **A** is represented the thalamic distribution of labeled cells following two injections in A1 (CT11 FB and DY). **B**. Alkaline phosphatase staining of an adjacent section. **C**. Density profiles showing the number of labeled neurons observed on regular serial sections across the LP. The distance axis corresponds to the position of the individual sections on the latero-medial dimension of the brain. The LP projection to A1 is relatively weak, as shown by the paucity of retrogradely number of labeled cells.

The different thalamic and cortical areas were identified using differential histological staining patterns. Phosphatase alkaline and cytochrome oxidase activity associated to Nissl coloration permit to find the limits between many areas. For example, in the thalamus, the location of the ventral nuclei of the medial geniculate body (MGv) was defined using multiple criteria. A dark CO and NAP background staining labels the latero-ventral area that correspond to the MGv. In Nissl staining, the MGv presents a biggest neuron density compare to the others one. All of these patterns are illustrated in figures 4–6. In order to determine the localization of the injection sites, we used similar multiple criteria to define the limits between cortical areas including CO, NAP and Nissl staining and completed by the sulcal position (see figure 1–3). For example, the limit between A1 and the secondary auditory cortex (A2) was identified using the difference of the AP and CO background staining of the layer 4, which is darker in A1 compared to A2 (Fig 3C). A similar difference was observed when comparing A1 and DZ as in the later area the AP and CO staining in the layer 4 is weaker compared to that observed in A1 (Fig 3A). Finally, SMI-32 provides further distinction of the DZ/A1 border [35] based on the density of staining in the supragranular layers as well as the presence of large stained cells in layer V of DZ (Fig 3B and 3G). Using the combination of these staining techniques, the location of stained neurons and the position of the injections sites could be localized.

## Results

The aim of the present study was to analyze the changes in the connectivity pattern of the auditory cortex induced by a congenital deafness. With the injection of retrograde dyes we targeted two main auditory areas, the dorsal zone and the primary auditory cortex A1. These two target areas were selected based on the behavioral study performed in deaf cats [12]. Compared to the primary auditory cortex A1, which is now well functionally defined (see [39] for review), less is known about the functional organization of the dorsal zone DZ. In cat DZ was initially designated as the dorso-posterior area DP, dissociated from A1 based on its absence of clear tonotopic organization [40]. The few electrophysiological studies performed described DZ neurons with broad frequency tuning and complex temporal firing properties [41]. A recent study suggests that DZ could be specifically involved in spatial localization to near-midline targets [42].

### Injection sites

This study is based on 16 injections of different retrograde dyes performed on 4 cats, 2 NHCs and 2 CDCs. After careful analysis of the injection sites, 14 injections were used for the connectivity analysis and 2 were excluded from further analysis because they were too small to provide reliable assessment of the cortical connectivity.

#### DZ injections

The area DZ is located dorsal to A1 and extends approximately 75% of the total distance into the supra-sylvian sulcus SSS [43], [44]. We attempted to avoid very deep injections within the SSS so as to prevent a contamination of the area ALLS (anterolateral lateral suprasylvian area) located ventrally in fundus of the sulcus [45]. All the injections sites are described in Table 1 and figures 1–3.

In NHC, three restricted injections of retrograde dyes were performed in DZ, which are all illustrated in figure 1 G–H. In CT10-FR, the location was assigned to DZ. The site was dorsally close to the SSS and rostrally at half distance between the AES and PES. The site covered the cortical thickness but it encroached in the underlying white matter for less than 20% of it total length. Concerning the cases CT15-FB and CT15-FE (see fig 1H and 3D), the injections were identified in DZ and were similar to the previous one. The sites covered all laminar layers and were restricted to a region close to the cortical surface under the ventral circumvolution of the SSS. No contamination of the white matter was observed.

Three injections in the deaf cats were allocated to DZ. The injection CT16-FE was defined as a restricted DZ injection (Fig 2H). It was located ventrally to the SSS at 1 mm distance. This injection was small but covered all laminar layers. The figure 3 presents sections reacted for CO activity (3A) or stained for SMI-32 (3B) and confirms the restriction of this injection to area DZ without a clear encroachment into A1 or into the area ALLS located down in the sulcus. CT11-FE was also allocated to DZ, but involved the white matter for about 40% of the antero-posterior length (Fig 2G). In the antero-posterior axis, this injection was located caudally. Because the retrograde dye injection was restricted to upper part of the supra-sylvian sulcus, the involvement of area PLLS is improbable and if present at all it would have been very small. Concerning the last DZ injection case CT11-FR (Fig 2F), the injection site was defined as DZ mainly. It was positioned at 1 mm ventrally to the SSS and covered all laminar layers. In the rostrocaudal orientation the site was located rostrally at the half of the distance AES-PES. This injection covered all the layers from the cortical surface and the labeling disappeared deep in the sulcus close to the border between DZ and ALLS.

#### A1 injections

We injected retrograde dyes in A1 in 3 cases for NHC and 2 cases for CDC (see Table 1 and figures 1 and 2). Paired of parallel injections were performed in order to analyze the topographical organization of the thalamo-cortical projecting neurons as it provides important information on its nucleotopic organization.

In NHC, case CT10-FB, the FB injection was clearly within the cytoarchitectonic boundaries of primary auditory cortex A1 (Fig 1E, 3F) and involved all the thickness of the cortex, from pia to white matter near the injection deposit center. It was located at 3 mm ventrally to the SSS and at half distance of the AES-PES region. In the same animal, the retrograde injection covering all layers (CT10-DY) was anterior and adjacent to the previous one and close to the AES (Fig 1D), suggesting a possible small contamination of the Anterior auditory field (AAF). The third A1 injection (CT15-FR) was similar to the CT10-FB in term of its antero-posterior position but it was positioned more dorsally (2 mm ventral from the SSS, Fig 1F). As shown in figure 3C in a CO stained section, the injection was adjacent to the A1/DZ border without a contamination of the auditory dorsal zone DZ. All these injections including their size and location with respect to the main sulcis (AES and PES) are illustrated in Fig. 1 (D–F).

In CDCs we have considered 2 injections targeting A1. In case CT11-FB (Fig 2D), the core of the injection was located at 3 mm ventrally to the SSS and at the half of the distance AES-PES. Close examination of the pick-up zone suggests that a small encroaching of DZ cannot be excluded. All laminar layers were covered by the injection. In the case CT16-FR, the injection site was located in A1 and was adjacent to the previously described injection (Fig 2E). The injection was located at 1.5 mm ventrally to the SSS but at the level of the AES and thus it was located at the anterior limit between A1 and AAF. The SMI 32 staining (figure 3G) revealed that the injection site did not spread into the area DZ. All laminar layers were covered. The reconstructions on serial sections of these two injections sites are illustrated in figure 2 D–E.

#### Other injections

Three of the 14 injections performed in both CDC and NHC were not included in the quantitative analysis because their location was overlapping into two other areas. These 3 injections are described in the Supplementary Material S1 (Sup. Table 1–4 in S1). One injection in the NHC targeting DZ (CT10-FE) was entering deep into the sulcus and involved both DZ and ALLS. In the CDC, one injection (CT11-DY) was located anteriorly at the level of the AES and was assigned to both AAF and A1, while the case CT16-DY was in its majority located to AAF with some involvement of A1. These last 3 injections will be, however, used in the present study for specific illustration of the topographical organization of the connectivity pattern (see discussion and Supplementary Material S1). However, the labeled neurons resulting from these 3 injections are not included in the quantitative analysis.

**Table 2:** Quantitative analysis of the strength of projection to A1 and DZ with respect to the total number of cortical inputs.

To A1	MGv	MGd	MGm	Po	SG	VB	LP	Pul						
Lee & Winner	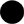	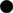	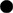	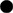										
NHC	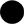	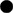	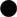	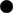	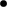	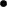								
CDC	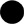	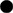	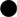	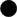	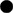	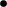	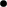	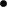						
To DZ	MGv	MGd	MGm	Po	SG	VB	LP	Pul						
Lee & Winner 2008	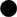	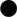	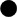	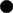	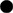									
NHC	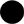	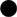	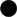	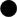	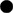	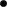								
CDC	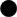	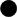	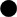	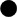	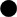	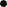	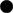							
To A1	A1	AAF	P	VP	Ve	A2	AES	DZ	Te	In	ED	EI	Ev	Others
Lee & Winner		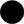	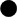	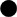	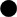	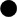	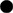	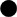	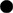	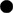	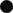	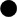	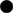	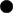
NHC		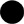	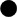	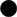	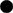	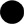	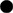	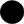	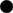	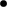	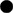	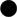	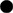	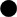
CDC		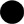	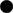	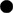	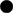	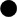	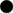	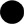	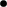	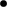	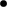	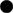	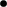	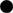
To DZ	A1	AAF	P	VP	Ve	A2	AES	DZ	Te	In	ED	EI	Ev	Others
Lee & Winner	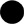	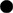	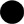	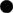	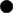	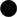	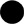		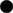	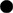	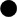	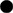	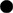	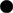
NHC	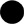	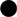	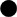	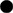	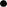	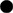	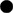		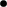	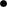	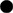	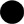	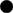
CDC	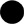	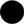	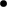	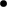	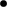	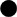	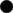		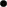	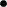	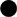	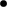	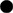	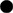
>20% total	Between 5-20% total	Between 1–5% total	<1% total	>20% total	Between 5–20% total	Between 1–5% total								
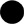	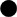	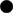	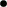	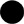	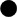	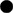								

The data obtained in the present study in the normal (NHC) and deaf (CDC) cat are compared with the previous study of Lee and Winner 2008.

**Table 3:** Thalamic labeling. The number of neurons and the number of sections sampled are indicated.

		*A1 injections*										*DZ injections*											
Group		NHC						CDC				NHC						CDC					
*Case*		*CT10 FB*		*CT10 DY*		*CT15 FR*		*CT11 FB*		*CT16 FR*		*CT10 FR*		*CT15 FB*		*CT15 FE*		*CT11 FR*		*CT11 FE*		*CT16 FE*	
Number of Neurons and sampled Sections		Nr	Sct	Nr	Sct	Nr	Sct	Nr	Sct	Nr	Sct	Nr	Sct	Nr	Sct	Nr	Sct	Nr	Sct	Nr	Sct	Nr	Sct
	MGD	8	7	14	2	42	5	391	12	139	6	73	10	51	5	93	7	73	12	127	10	94	7
	MGM	18	3	44	5	576	16	387	13	502	18	88	13	40	8	103	8	251	15	270	19	42	5
	MGV	1399	17	1175	17	705	16	3131	19	601	12	381	15	61	12	215	14	65	9	333	17	146	10
	Po	10	2	4	1	99	5	22	5	483	8	46	7	50	9	146	9	331	13	288	15	39	8
	SG					18	7	8	2	120	11	15	8	11	3	13	1	307	13	113	11		
	VB					29	17	3	2	19	9	1	1	1	1			13	3			1	1
	LP							27	6	8	6									81	14		
	Pul									1	1												
**Thalamic labelling**		**1435**		**1237**		**1469**		**3969**		**1873**		**604**		**214**		**570**		**1040**		**1212**		**322**	

**Table 4:** Cortical labeling. The number of neurons and the number of sections sampled are indicated.

		A1 Injections										DZ Injections												
Group		NHC						CDC				NHC							CDC					
*Case*		*CT10 FB*		*CT10 DY*		*CT15 FR*		*CT11 FB*		*CT16 FR*		*CT10 FR*		*CT15 FB*			*CT15 FE*		*CT11 FR*		*CT11 FE*		*CT16 FE*	
Number of Neurons and Sections Used		Nr	Sct	Nr	Sct	Nr	Sct	Nr	Sct	Nr	Sct	Nr	Sct	Nr	Sct	Nr		Sct	Nr	Sct	Nr	Sct	Nr	Sct
	**Areas**																							
Tonotopic	A1	///	///	///	///	///	///	///	///	///	///	3298	21	125	8	684		8	1019	17	719	17	338	9
	AAF	125	6	3022	8	584	3	486	8	1387	11	588	6	12	3	194		4	133	7	59	7	580	6
	P	48	3	240	4	344	5	153	4	38	4	17	3	187	3	186		3	11	5	36	5	1	1
	VP	52	3	558	3	114	5	93	5			3	2	43	3	75		3	2	5	39	5	1	1
	Ve	5	2	38	3	125	2	105	5	0	2	5	3						8	2	21	2		
***Number of neurons***		***230***		***3858***		***1167***		***837***		***1425***		***3911***		***367***		***1139***			***1173***		***874***		***920***	
Non Tonotopic	A2	201	9	2035	10	587	8	244	10	115	8	374	10			3		1	254	12	323	12	6	3
	AES	19	5	284	10	45	4	38	8	28	3	139	9	37	3	33		7	201	9	79	9	2	1
	DZ	131	13	1992	13	906	9	975	17	1145	13	///	///	///	///	///		///	//	///	///	///	///	///
***Number of neurons***		***351***		***4311***		***1538***		***1257***		***1288***		***513***		***37***		***36***			***455***		***402***		***8***	
Limbic	Te	48	7	357	8	17	5	14	4	0	2			9	1				20	8	40	8	1	1
	Insula	5	3	42	7	23	8	42	8	1	6	11	5	2	1	1		1	46	15	26	15		
	35/36							25	9										43	26	60	26		
***Number of neurons***		***53***		***399***		***40***		***81***		***1***		***11***		***11***		***1***			***109***		***126***		***1***	
Temporal	ED	59	6	103	7	67	5	2	2	10	3	57	6	49	7	8		2	440	14	369	18	27	5
	EI	18	3	207	8	355	7	42	4	5	1	504	7	235	6	219		5	287	11	414	11	19	4
	Ev	14	4	161	8	22	6	9	3	5	4	39	6	14	2	5		3	502	19	129	19		
***Number of neurons***		***91***		***471***		***444***		***53***		***20***		***600***		***298***		***232***			***1229***		***912***		***46***	
Parietal SSS	ALLS	113	14	508	15	35	5	92	9	65	11	295	14	16	2	31		4	206	8	67	8	53	2
	AMLS	2	2	5	1	1	1			1	2	3	3			1		1	54	7	2	7		
	PLLS	1	1	2	1	49	7	1	1	12	3	21	4						774	26	134	26	13	5
	PMLS					10	1			0	1								172	17	6	17	1	1
***Number of neurons***		***116***		***515***		***95***		***93***		***78***		***319***		***16***		***32***			***1206***		***209***		***67***	
Posterior Ectosylvian Gyrus	19																		15	8	6	8		
	20									5	5								159	31	75	31	2	2
	21																		48	7	13	7		
	PS					3	1			2	2								4	1	4	1		
***Number of neurons***						***3***				***7***									***226***		***98***		***2***	
Anterior AES	VAE																		9	10	21	10		
	S-IV																		4	5	8	5		
***Number of neurons***																			***13***		***29***			
Somato-motor	4										1								2	3	2	3		
	5					2	2			1	3	9	8			3		3	9	7	4	7		
	6																		13	7	4	7		
	7					3	1					3	3	4	2	8		5	332	34	13	34	5	2
	S2					1	1	4	2										3	3	2	3		
***Number of neurons***						***6***		***4***		***1***		***12***		***4***		***11***			***359***		***25***		***5***	
Others	PFC					2	2			5	4	10	6	2	1	4		4	75	16	43	16		
	CG					2	2			0	1	12	8	6	2	11		6	73	28				
***Number of neurons***						***4***				***5***		***22***		***8***		***15***			***148***		***43***			
***Total Labeling***		***841***		***9554***		***3297***		***2325***		***2825***		***5388***		***741***		***1466***			***4918***		***2718***		***1049***	

### Distribution of projecting labeled cells in thalamic nuclei and cortical areas

We mapped the distribution of the retrogradelly labeled cells in the thalamic structures using the anatomical parcellation described by Imig and Morel [46], [47], see also [48], [49] for reviews). The aim of this study is to detect abnormal connectivity in CDC.

### Thalamic projections

#### Thalamic inputs to A1

Dye injections in the primary auditory cortex A1 lead to a thalamic distribution of projection cells mainly in the medial geniculate body in normal hearing cats, as previously reported [29], [46], [47], [50], [51]. Here we found in the CDCs that thalamic cells projecting to DZ were primarily located in the MGB (medial geniculate body). A global inspection of the cell distribution revealed a similar pattern of thalamic projections in both groups (Fig 4A and Fig 5 and table 2). The ventral subdivision of the MGB (MGv) constitutes the predominant nucleus projecting to A1 in both the CDC and NHC (Table 3; Fig 5) in agreement with previous quantitative studies in normal hearing cats [29]. The remaining projections are originating principally from the dorsal and medial MGB subdivisions and from the lateral part of the posterior thalamic nuclei, PO.

Analysis of the distribution of labeled cells indicates some disparities between the normal and the deaf cats with a tendency for a lower inputs density arising from the MGv and a stronger projection from PO in the CDC (Table 2). More importantly, in the CDCs we observed a specific projection from a visual thalamic nucleus, the lateral posterior (LP), which was not found in controls. This projection was observed for the two A1 injections in the CDCs (Fig 6). The projection was relatively weak, representing less than 1% of the overall thalamic inputs to A1 in the deaf cat. However, the projection zone was topographically well organized in the antero-posterior axis – the labeled neurons were not randomly scattered across the nucleus. Instead, the density profile of the labeled neurons showed the bell shape characteristics of a classical projection [37]. This projection may provide a weak non-auditory input directly to the auditory cortex of the deaf animals.

In each group, we have performed paired dye injections in the primary auditory cortex in two frequency representations. In the NHC (CT10), the FB-DY separation into A1 is of 1.6mm, while it is slightly greater in the CDC (CT11, 2.3mm). These separations, which correspond to the distances between dyes deposit centers, represent approximately a separation of approximately two octaves according to the tonotopic organization of the cat A1 [28], [51]. Because of the tonotopic organization of the MGv [46], this double injection resulted in a segregation of the projecting neurons in the thalamic nucleus as illustrated in the case of control cat (CT10 FB-DY, Fig 5A). Similarly, in the CDC a double injection performed in the middle (CT11-FB) and high frequency representations (CT11-DY, partially spilling over into AAF) resulted in a complete spatial separation of the projecting cells in the MGv, demonstrating a preserved nucleotopic organization. However, the projections were more spread than in NHC, corresponding to a functional smear of the cochleotopic gradient (compare [52], [53]). The complex topography of the distribution of labeled cells reflects the complexity of the frequency representation along the 3D dimension of the MGv [44], [46].

#### Thalamic inputs to DZ

In both normal and congenitally deaf cats the distribution of the thalamic projections directed toward DZ can be distinguished from that reported following an A1 injection. As previously reported [29], the DZ projections are more equally distributed across the different subdivisions of the MGB, which constitutes the principal source of projection toward the area DZ (Tables 2 and 3). Again the patterns of distribution were similar when comparing the controls and deaf cats (Fig 4B), including a strong projection arising from the posterior nucleus PO (above 20% in both groups). A specific set of retrogradely labeled cells in the visual thalamic nucleus LP was again found exclusively in deaf cats.

### Cortico-cortical connections

**Figure 7:**
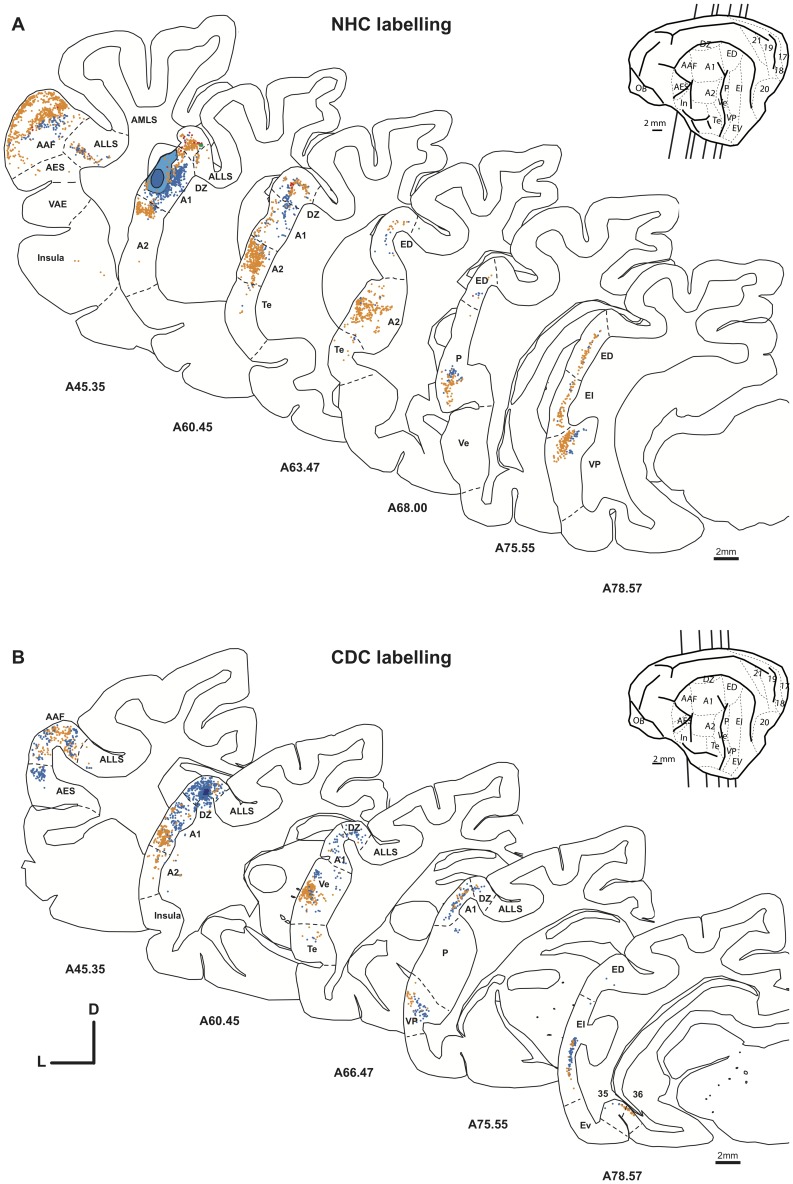
Distribution of the projecting neurons to A1 following a FB (blue) or a DY (yellow) injection in the normal (A, case CT10) and in the deaf (B, case CT11 FB and DY) cat. Only selected sections are shown to illustrate the distribution of labeled cells in the various auditory areas. The insert shows a schematic view of a cat brain areas and the location of the illustrated sections in the antero-posterior axis. Conventions as in figure 5.

**Figure 8:**
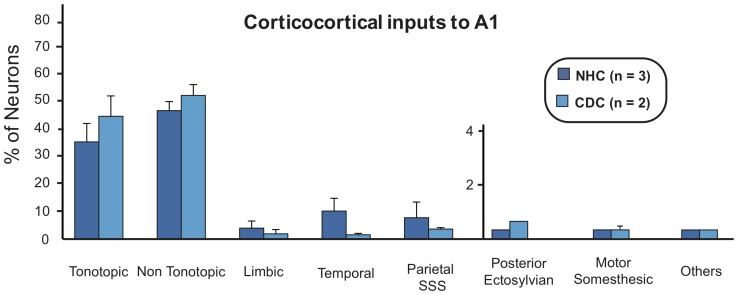
Histograms of the means of the distribution of cortical projecting cells following an injection in A1 in the normal (dark) and deaf (light) cat. The cortical regions that are included in each classes are provided in table 4. For simplicity and because of the low number of labeled cells, projection from the AES and the somato-motor regions are grouped together in the “motor somesthesic” group. The pattern and density of projections are similar when comparing both normal and deaf cats. Conventions as in figure 4.

**Figure 9:**
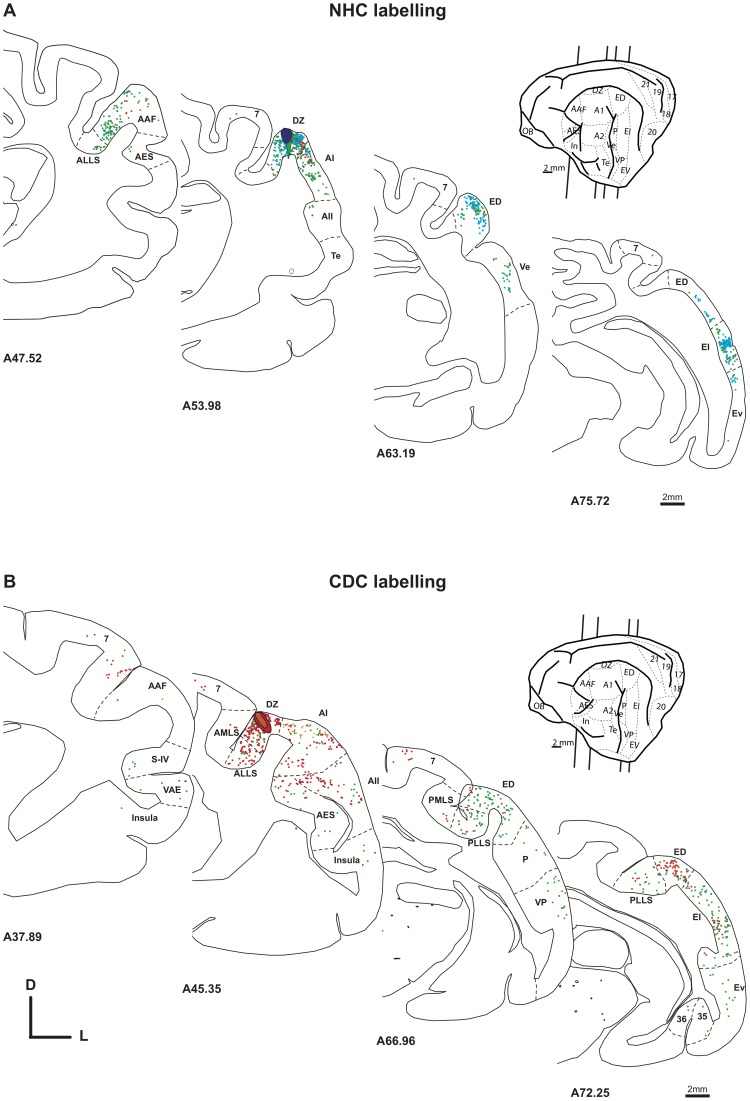
Distribution of the projecting neurons to DZ in the normal (A, case CT15 FB and FE) and deaf (B, Case CT11 FE and CT11 FR).) cat. Convention as in figure 7.

**Figure 10:**
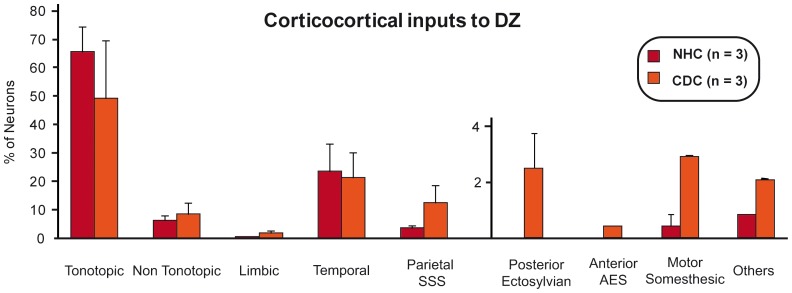
Histograms of the means of the distribution of cortical projecting cells following an injection in DZ in the normal (dark) and deaf (light) cat. Convention as in figure 4 and 8.

The cortical auditory system of the cat is composed of several areas that can be defined anatomically by specific cytoarchitecture and connectivity patterns as well as electrophysiologically based on the frequency representation (tonotopic organization). In the present study we used the subdivisions of the auditory areas according to the recent work of Winer and colleagues [54]. For simplicity in the illustration, the charted neurons projecting to A1 (Fig 7) or DZ were grouped together into large classes of source areas (e.g. tonotopic and non-tonotopic, Fig 8, Fig 9 and 10), but the individual counts in the cortical regions are provided in table 4. Concerning the non-auditory areas, we have used the cortical parcellation of Scannell and col. [55].

#### Cortical inputs to A1

Following dye injections in the primary auditory cortex A1 of both controls and deaf cats, we observed a dense retrograde labeling throughout a large extent of the auditory cortex. In the rostro-caudal axis, projecting neurons were observed from the auditory anterior field (AAF) to the posterior ectosylvian gyrus. In both groups, labeled cells were observed dorsally in the DZ area as well as in the ventro-posterior auditory area (VP). Figure 7 illustrates the distribution of retrogradely labeled cells in serial sections distributed across the brain of a control (A) and a deaf cat (B). In both groups, the tonotopic (AAF, P, VP and Ve) and non-tonotopic (A2, AE, DZ) auditory areas constitute the principal inputs to A1 as they contain over 80% of the total of projecting cells (Fig 8). In both the CDCs and the NHCs, the bulk of projections to A1 originate from a limited number of auditory areas among them the anterior auditory field AAF, A2 and the dorsal zone DZ (Tables 2 and 4). Such pattern of auditory projections to A1 is highly consistent with that demonstrated in normal hearing cats (e.g. [56]). Outside theses main projections, A1 is receiving sparse inputs from various areas of the temporal cortex (TE, IN), of the polymodal posterior ectosylvian gyrus (ED, EI) and of the visuo-auditory areas buried in the suprasylvian sulcus (areas ALLS, AMLS, PLLS, PMLS). In theses later cases, the individual proportions originating from a specific area might vary when comparing the NHCs and CDCs, but the limited number of cases precludes the application of statistical tests on these differences.

All together, the comparison of the connectivity pattern of A1 in CDCs did not substantially differ from that observed in the controls. We did not observe any abnormal cortical projection to A1 in deaf cats that was not present in the control group and has not been previously reported in the literature.

#### Cortical inputs to DZ

We performed three injections of dyes in each group of cats. In both the normal and deaf cats we found the majority of inputs to DZ were located in the tonotopic auditory areas. These projections constituted over 50% of the total of afferents to DZ, especially from the areas A1 and AAF [43], [56], [57]. Further, the posterior areas ED, EI and EV composed the second largest set of cortical projections to the dorsal zone. In consequence, as illustrated by the distribution of charted projecting neurons across the auditory areas (Fig 9) as well as the comparison of strength of projection (Fig 10, Tables 2 and 4), the connectivity patterns of DZ were rather similar between the deaf and the normal cats.

However, we observed some consistent novel projections to DZ in deaf cats not present in normal hearing cats. These “abnormal” connections arose from areas that are considered either visual or somatosensory, but are not associated to the auditory system. All together, these abnormal projections in the deaf cat represent less than 6% of the total of inputs to DZ and the individual contribution of specific areas is quite low. Among them, the visual areas of the ventral posterior ectosylvian gyrus constitute the main source of abnormal projections to DZ in the deaf cat.

**Figure 11:**
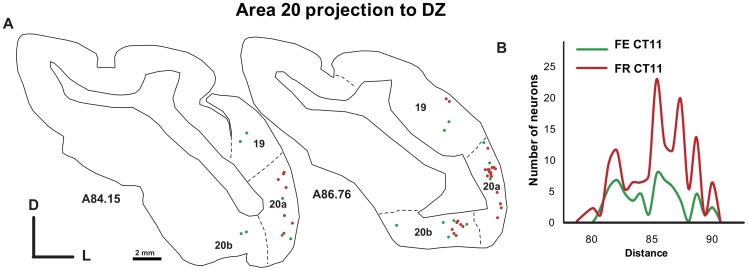
Abnormal visual projection from the areas 19/20 to area DZ in the deaf cat (Case CT11 FE and CT11 FR). In A is shown the distribution of projecting cells in two adjacent sections. The density profiles are presented in B. Convention as in figure 6.

In deaf cats, novel topographically organized cortico-cortical connections from non-primary sensory areas 19-20a-20b/21 to the DZ were observed (Fig 11, table 4). It is important to note that in the CDCs, the injections of dyes in A1 did not lead to labeled neurons in these areas, making these projections specific to DZ. No labeled cells were observed in these visual regions of normal cats, neither after DZ nor A1 injections. In addition, in the normal cat, one DZ injection was analyzed separately because it was largely spanning into the visual area ALLS in the fundus of the sulcus (case CT10-FE, Supplementary Material S1, Sup. Table 3). In this case, we did not observe a projection from the areas 19 and 20a/20b. Thus, methodological reasons for this projection are unlikely. The weight of this reorganized visuo-auditory pathway is weak, but it also does not correspond to scattered projecting neurons, as can be seen from the topographical organization of the charted neurons (Fig 11). The projection zone shows the typical density profile of a cortical projection with a bell-shaped peak of density in the center and minimal values in the periphery [37].

**Figure 12:**
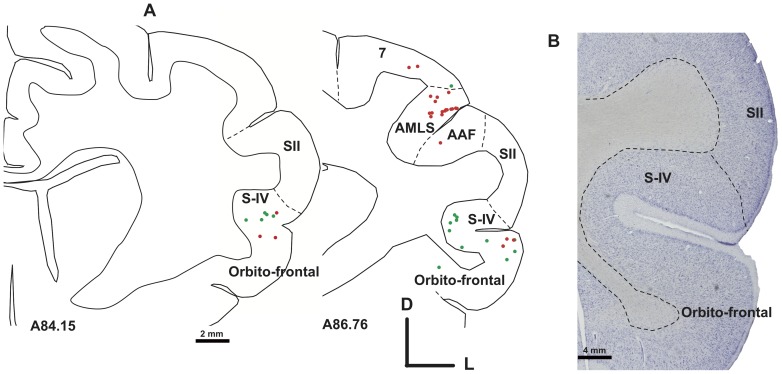
Illustration on two representative sections of the presence of an abnormal projection from the somatosensory area S-IV to DZ in the deaf cat (Case CT11 FE and CT11 FR). The panel B is a Nissl staining of an adjacent section. Note the high number of labeled cells located in the parietal area 7.

A second set of novel topographically organized cortico-cortical connections to DZ in deaf cats originated from non-primary areas AMLS and SIV. The dorsal rim of the anterior end of the anterior ectosylvian sulcus (AES) hosts the somatosensory area SIV. It is located ventrally to the secondary somatosensory area (SII, [58]). We observed retrogradely labeled neurons in the area SIV of the deaf cat following the DZ injection (Fig 12). In addition, labeled neurons were observed further ventrally below the area SIV in a region that might encompass the para-SIV subdivision [58] and the multisensory orbito-insular region [59]. In normal hearing cats, the three injections performed in DZ did not reveal any projecting neurons in this somatosensory area. Again, the weight of this abnormal projection in the CDCs was low and represented less that 1% of the total of afferents to DZ.

Finally, we observed some non-auditory projections to DZ that originated from the middle suprasylvian gyrus, a cortical region corresponding to the visuomotor parietal area 7 [60], [61]. Such projection was present in both groups of animals. However, the strength of projection in the controls was negligible, as we observed only few scattered projecting neurons. Many more cells in areas 7 and AMLS were connected to the DZ in the CDC animals as compared with normal hearing animals. As the injections sites were comparable across the two groups, it is probable that the increased number of projecting neurons from the parietal cortex to DZ is an additional specific feature of the deaf cat.

#### Callosal connectivity

We have analyzed the distribution of the projecting neurons located in the hemisphere contralateral to the injections sites. For both A1 and DZ we observed that the majority of inputs arose from the homotopic areas (Table 5). Following the injections in A1, we observed a predominant proportion of labeled cells in the contralateral A1 (controls: 70%; deaf: 65%). The callosal connectivity of A1 originated predominantly from the tonotopic auditory areas (90% in NHCs and 80% in CDCs). No specific difference was observed between the normal and the deaf cats.

**Table 5:** Callosal cortical labeling. The number of neurons and the number of sections sampled are indicated.

	A1 Injections										DZ injections											
*Group*	NHC						CDC				NHC						CDC					
Case	CT10 FB		CT10 DY		CT15 FR		CT11 FB		CT16 FR		CT10 FR		CT15 FB		CT15 FE		CT11 FR		CT11 FE		CT16 FE	
Number of Neurons and Sections Used	Nr	Sct	Nr	Sct	Nr	Sct	Nr	Sct	Nr	Sct	Nr	Sct	Nr	Sct	Nr	Sct	Nr	Sct	Nr	Sct	Nr	Sct
A1	149	13	376	12	65	2	237	14	53	9	45	8	2	1	6	3	27	9	51	10	1	1
AAF	12	3	6	2	9	2	1	1	26	6	9	3					11	4	9	5	2	1
P					1	1	46	1		1			4	2					3	1		
VP							1	1		1												
Ve										1			1	1					4	2		
A2	8	5	63	5			7	1		3	1	1	1	1			15	3	12	6		
AES	1	1			4	2				1	6	2					3	2	2	2		
DZ	5	4	37	10	52	3	52	12	19	10	54	12	18	5	23	4	119	13	29	9	9	4
Te			2	1															1	1		
Insula																	7	4	3	2		
35/36							4	2														
ED	1	1					2	2		5	4	1					1	1	2	1		
EI	1	1					1	1		4							3	1	2	2		
Ev									2	1												
ALLS	1	1	13	3	1	1				2	7	4					73	13	1	1		
AMLS																	3	3				
PLLS										5							18	6				
PMLS										2							3	1				
20									2	4												
PS									1	1												
***Total Labelling***	178		497		132		351		103		126		26		29		283		119		12	

Similarly, we did not observe abnormal patterns of callosal connectivity of the area DZ in the deaf cats. Also here, the majority of projections arose from the homotopic area DZ (48% in the CDCs vs. 64% in the NHCs). The only slight difference in the respective proportion was observed from the inputs originating from the area ALLS, which is slightly higher in the deaf cat (7% vs. 1% in the controls).

All together, we did not observe a specific abnormal pattern of callosal connection in the congenital deaf cats.

## Discussion

In the present study we have shown an anatomical reorganization of the connectivity of auditory areas DZ and A1 in congenital deafness. Specifically, the auditory DZ area of deaf cats receives non-auditory inputs from the multimodal SIV/orbito-frontal regions as well as from the visual areas 19/20a-20b, and more projections from AMLS and area 7. Further, we have observed abnormal non-auditory inputs from the visual thalamic nucleus LP to the primary auditory cortex A1. Such field specificity of reorganization is in accordance with previous work [12] that revealed a functional dissociation between A1 and DZ in crossmodal reorganization after deafness.

In addition, the present study demonstrates that these abnormal projections represent only a small fraction of total inputs. The large majority of the afferents to A1 and DZ appeared normal in their areal specificity.

### Reorganization of the connectivity of areas A1 and DZ of the congenital deaf cat

The tracer injections in both A1 and DZ revealed a limited set of abnormal projections present only in the CDC. This abnormal connectivity originates from structures involved in visual or somato-motor processing and could provide non-auditory information to the deprived auditory system.

#### Thalamic inputs to the primary auditory cortex A1

A direct comparison revealed that the thalamic sources of inputs to A1 after congenital deafness are close to that observed in normal hearing cats, namely with a major projection from the ventral subdivision of the MGB. The area A1 showed only a single source of abnormal afferent which corresponds to the thalamic nucleus LP. Further, the strength of projections of individual thalamic pathways in the CDC were close to that of normal hearing cats (see tables 2-5). Additionally, the nucleotopic organization in the thalamo-cortical pathway was grossly preserved in the CDC. The double injections of dyes in two isofrequency bands of A1 lead to a segregated population of projecting neurons as observed in the normal hearing cats [44], [62], [63] or in partially deafened cat [64]. Nonetheless, the spread of the dye was larger following congenital deafness, indicating a functional smear of the cochleotopic gradient (compare [52], [53]). This suggests that a congenital deafness does not completely destroy the nucleotopic organization of the thalamo-cortical projection and that this organization is not the consequence of plastic adaptation to auditory input. However, this observation should be taken with precaution, given the complexity of the frequency representation in the 3D representation of the MGB [46], [65], [66]. In addition, electrophysiological recording of congenitally deaf cats by mean of electrical stimulation revealed an abnormal pattern of activity in A1 [67] corresponding to a loose cochleotopic representation [52] for neonatally deafened cats (see [68] but compare [53], [69], [70]. A larger spread could be caused by a higher divergence of the arborization of the thalamo-cortical projecting neurons [71] and a reduced intrinsic cortical inhibitory mechanism (ibid).

We observed a single source of non-auditory thalamic inputs to A1 in the deaf cat. The thalamic nucleus LP (lateral-posterior) is projecting directly to A1. This nucleus is part of the cat LP/pulvinar complex that is considered as a visual thalamic relay [72]. The location of the LP projecting neurons could correspond to the LPm subdivision according to the previous LP segmentation based on anatomical [73] and electrophysiological grounds [74]. The visual responses recorded in the LP nucleus [75] derived probably from its visual inputs arising directly from the superficial layers of the superior colliculus and from various visual areas of the suprasylvian sulcus [76], [77]. Of interest is that in animal models where selective neuronal lesions early in life induced a *rewiring* of the brain, LP projections have been identified to reach the primary auditory cortex [78] that had acquired visual functions [79]. It is possible that the lack of auditory activity reaching AI early during development makes this area more susceptible to be invaded by direct projections specifically from the visual LP nucleus.

These inputs from the LP, in addition to a trend for a higher density of projection from the SG nuclei, contribute probably to the transmission of non-auditory information (mainly visual) to the auditory cortex of the deaf cat.

#### Cortical inputs to area DZ

While we did not observe any novel cortical projection to A1 in the deaf cat, we found a consistent projection to area DZ of the CDC from the visual areas of the posterior suprasylvian gyrus including areas 19/20/21. As developed further (see methodological considerations), areas 20 and 21 are connected with the dorsal visual areas in the sylvian sulcus [80]–[83] for which we cannot rule out the possibility that they have been contaminated by our dye injections. The cortical areas 19/20a-20b are considered as purely visual, they contain a full representation of the contralateral visual field having neurons with large receptive fields [84], [85]. Indeed, behavioral studies in cats have implicated the areas 20/21 in visual learning of pattern discrimination [86]–[88]. It is consequently probable that the direct projections we observed from areas 20/21 to DZ contribute to the visual function supported the dorsal auditory zone.

The upper rim of the anterior part of the anterior ectosylvian sulcus was shown to project directly to area DZ in deaf cats but not in hearing controls. This region SIV, located anteriorly to the auditory and visual fields of the AES [89]–[91], is defined as a somatosensory area with a representation of the body surface distinct from the adjacent area SII [57], [58]. In consequence, the projection observed from SIV to DZ of the deaf cat could provide somatosensory information but also some visual information because some (but few) SIV cells respond to moving spots of light [92]. In addition we observed a consistent projection from the region located below the SIV area and corresponding probably to the orbito-insular frontal cortex. This area, which contains both visual and auditory responsive neurons [59], has been also implicated in visual behavior such as the localization of visual target in the peripheral visual field [93]. In agreement with the present results, previous studies on sensory deprivation from birth have emphasized on the implication of the cortical region buried in the AES in crossmodal plasticity [94], [95], but no work have yet studied the role in crossmodal compensation of its anterior part which is more devoted to somatosensory processing.

Finally, we observed a stronger projection of area 7 and AMLS into field DZ in deaf cats. This would provide a further route of visual inputs to DZ and would strengthen its visual reorganization.

#### Functional dissociation of A1 and DZ in the deaf cat

An important observation from this anatomical work is the dissociation in the deafness-induced reorganization of the connectivity of A1 and DZ. In the normal hearing cat, A1 and DZ can be functionally dissociated with respect to their implication in spatial auditory processing [96], [97]. A functional dissociation remains present when considering crossmodal reorganization ([12] and present study). Area DZ of the deaf cat receives non-auditory inputs from various sources but most exclusively from cortical regions. On the opposite, we were able to reveal only a weak thalamic origin of crossmodal afferents to A1. The lack of cortical influence from other sensory modality directly to A1 might restrict such functional reorganization. This is in agreement with the behavioral and electrophysiological results. Firstly, when area A1 is inactivated, the congenital deaf cats do not present any loss of their supra-normal visual skills for both visual localization or motion detection [12]. When DZ is transiently ”silenced“, the visual performances fall down to normal levels. Secondly, in contradiction to early work based on evoked potentials [27], a systematic study based on electrophysiological recording of single cells in A1 of the deaf cat failed to reveal visual responses [25], [26]. These results strongly suggest that the primary auditory cortex of the cat is in certain extent “resistive” to a functional colonization by other sensory modalities. Such hypothesis is in agreement with some human studies showing that in deaf patients the activation of the auditory cortex by visual stimuli is restricted to the in secondary auditory areas but does not encompass in A1 [10], [24].

#### Methodological considerations

The present study is based on 2 or 3 injections in individual cortical areas, and in each case the topological distribution as well as the density of labeling were quite similar across dyes and animals (see fig 4 and 8). Our strategy of performing large stereotyped tracer injections spanning all the cortical depth, coupled with a high frequency sampling of the sections across the brain insures a strong reliability of the results [37] by reducing the variability [98]. Further, recent work on the visual cortex connectivity in the monkey showed that the areal specificity and the strength of connections are relatively constant across animals [38]. Following the exploration of the entire projection zone [37] the consistency of a given cortical projection could be explored even based on a small number of cases as in the present study [38].

Some injections used here were not restricted to the cortical plate and encroached the underlying white matter. The influence of depth injection is problematic in developmental studies because cortical transient connections do not penetrate the cortical grey matter [99], [100]. However, in the adult. when the dye injections are invading the white matter, it has no effect on the areal specificity and the density of labeling [38], [101]. This is illustrated in cases CT-11 FR and FE, which, in spite of one of this injection located deep in the white matter (CT-11 FE), showed a similar pattern of labeling including the presence of abnormal projections from the visual areas. This suggests that the depth of dye injection is not responsible for the different pattern of connectivity observed between the normal and the deaf cats.

The main result of the connectivity analysis of A1 in the deaf cats is the absence of extensive abnormal cortico-cortical connectivity. In spite of the fact that our injections sites might not always being restricted to a single area, it is important to note that the overall pattern of distribution of retrogradely labeled cells in the CDC correspond to the one previously reported for A1 injections in hearing cats [56]. As previously observed in normal hearing cats [102]–[104], the main afferents to AI of the CDC originate from areas A2, AAF and the posterior temporal auditory areas (ED, EI, Ev). In any case, the possibility that the injection might have contaminated the area outside A1 cannot account for the lack of massive abnormal connectivity pattern of the primary auditory cortex of the CDC.

While we did not find any abnormal cortical projections to A1, the dorsal auditory area DZ had novel cortical connectivity patterns after congenital deafness. We cannot rule out the possibility that the visual inputs to DZ are resulting from a spread of the injections sites into the areas ALLS or PLLS because previous works have reported a weak and variable projection from the areas 19/20a–20b toward ALLS [80]. However, several observations suggest that a contamination of the injection in the area ALLS/PLLS does not account for the entire retrograde labeling in the non-auditory areas. First, no labeling was observed in the geniculate thalamic relay known to project to areas PLLS [105], [106], which suggests the absence of a significant contamination of the area ALLS/PLLS. Second, in the normal hearing cat we did not observe the abnormal projection from non-auditory cortical regions (areas 20/21, SIV) following a large injection encroaching the WM and contaminating the cortical area ALLS buried in the fundus of the SSS (Case CT10 FE, see Supplementary Material S1, Sup. Table 3). Third, in a late deafened cat (over 6 months after birth), it has been recently shown [107] that the area DZ is receiving a much higher density of visual projections, including projections from visual areas 19/20a–20b as reported presently. In consequence, it is probable that the visual inputs that reach the auditory area DZ in the deaf cat are representing a smaller proportion that the one reported presently here due to the possibility of a spread of the injection sites. Concerning the projection originating from the SIV and orbito-frontal region, no such projections have been described toward neither DZ nor toward the areas PLLS/ALLS [57], [80] suggesting that such projections are specific to the deaf cat area DZ.

### Role of afferent activity in the establishment of cortical connectivity

The present results provide evidence of a near normal connectivity pattern of the auditory areas A1 and DZ. This restricted impact of a lack of auditory activity during development is observed both at the levels of the thalamo-cortical pathway and the cortico-cortical network. Of importance, the basic functional architecture of the auditory cortex, the tonotopic organization, appears to be preserved to some extent. Altogether, our results question the role of sensory activity in governing the cortical connectivity. While the organization of some functional maps such as the ocular dominance columns in the primary visual fields V1 are largely dependent of the afferent activity (see [108]), anatomical works performed at early stages of the development [109], [110] have revealed a precise organization of the visual pathways before birth (see [111] for review). In particular, in monkey, the areal specificity and the hierarchical organization of the connectivity are present during the prenatal stages and no transient exuberant connections can be observed throughout the development [100], [101], [112]. These results are important for several reasons. First, they weaken the role of experience in pruning of exuberant immature inter-areal connections after sensory loss [113]. Such mechanism could nevertheless apply to rodents that present a poor precision in the formation of their connectivity pattern (see [114] for review). Second, it suggests that activity-independent mechanisms play an important role in the establishment of the connections before birth. In a primate model of congenital anophtalmia [115], [116] we have observed a near-normal areal specificity of the connectivity pattern of the visual areas V2 and V4 [117], [118], including a preservation of the retinotopic topology and a lack of crossmodal reorganization [119]. Altogether, this set of data, including the present work, supports the hypothesis that in high order species, a sensory deafferentation at birth cannot extensively alter the cortical connectivity.

### Crossmodal compensation and multisensory integration

Brain imaging studies have revealed abnormal metabolic levels [120], [121] and activations of the auditory cortex of deaf patients by visual or tactile stimuli [122], [123] that could originate from a direct functional impact of the non-auditory projections presently described. However, given the paucity of such abnormal projections, additional mechanisms should be considered to fully account for the crossmodal reorganization of the deafened auditory cortex. It might be possible that some reorganization of connectivity occurs before the thalamic level as observed in experimentally rewired brain [124]. Alternatively, crossmodal functional reorganization observed following congenital deafness might be supported, at least partly, by the normal network connecting the auditory system. Indeed, several sources of visual inputs can reach indirectly the auditory cortex and could participate to crossmodal reorganization. Visual or visual related (saccadic) information are reaching the different auditory relays such as the inferior colliculus and the MGB [125]–[127]. These visual activities can originate from direct retinal inputs [128], [129] or from projections from the upper layers of the superior colliculus [130] reaching the inferior colliculus. In some species (rodents and ferrets), the primary auditory cortex is receiving direct projections from visual areas [131], which is not the case in the cat and monkey (present study, [132]. For example, in areas of the auditory core of the ferret sparse but direct inputs from visual areas exist (including the primary visual cortex) that probably account for the small proportion of neurons that can be driven or modulated by visual stimulation [133].

This suggests that in some species such as the cat or monkey, the cortical mechanisms for crossmodal compensation after sensory loss can rely on unmasking or on an increased efficiency of existing heteromodal connections linking the auditory cortex to cortical or thalamic structures involved in processing visual or tactile modalities (see [17], [134]–[136], reviewed in [137]. Indeed crossmodal reorganization after total deafness can occur during adulthood: in the adult deafened ferret, somatosensory responses can be revealed in A1 neurons in absence of a reorganization of the connectivity of the auditory cortex [138]. Similarly in normal adult subjects, a sensory deprivation (complete blindfolding) can rapidly induce an engagement of the visual cortex including V1, in processing tactile information [139], adding further evidence that crossmodal compensation and multisensory integration can share a common anatomical pathway.

## Conclusions

In summary, in the congenital deaf cat, the auditory areas A1 and DZ receive abnormal visual and somatosensory projections not present in hearing cats. However, the strengths of these projections are weak, suggesting that crossmodal compensation after sensory loss results from the complementary contribution of a reorganized cortical connectivity and the normal network involved in multisensory processing. Ultimately, these results weaken then role of activity dependent mechanisms in the establishment of cortical connections during the development.

## Supporting Information

Supplementary **Material S1.**
(DOC)
