# Supplementary material for: Reorganization of the Connectivity of Cortical Field DZ in Congenitally Deaf Cat
Source: PLoS One. 2013 Apr 12;8(4):e60093. doi: 10.1371/journal.pone.0060093 (PMC3625188; doi:10.1371/journal.pone.0060093)
Supplement: Supplementary — Material S1. (DOC) [file pone.0060093.s001.doc]

**Supplementary material S1.**

We have discarded from the main analysis 3 injections which were not restricted to a single area and which were not directly comparable to the main injections performed in A1 and DZ.

In a normal hearing cat the injection (CT10 FE) was located in DZ but it was also spanning largely in the adjacent cortical area ALLS. In this case the thalamic labeling was similar to that observed in the other cases of DZ injections with a repartition among the 3 main thalamic nuclei. Note that some labeled cells are observed in the LP nucleus probably because of the large spread of the injection into the visual area ALLS which is known to be interconnected with the medial division of the LP [1,2]. The distribution of cortical labeling was close to the one obtained following a DZ injection but with a much stronger projection originating from the cortical areas of the lateral suprasylvian sulcus (areas AMLS, PLLS and PMLS) compared to that observed in case of a restricted DZ injection (8% vs 0.5% respectively). Of importance, no labeled cells was observed in the visual areas 19/20/21 confirming that these abnormal projections to DZ observed in the deaf cats were not due to any encroaching in the area ALLS.

In the deaf cat two injections aimed on A1 were rejected because they were largely spanning on the adjacent area AAF (cases CT11-DY and CT16-DY). In both cases, we observed a small abnormal projection from the LP nucleus as in the other CDC cases of restricted injection into A1. In the normal hearing cat, not such LP projections have been described to target the area AAF [3]. Altogether, it reinforces the observation of an abnormal visual inputs from the LP nucleus to A1 in the deaf cat. At the cortical level, we observed some labeled projecting neurons in S2 not present in the other cases. Further experiments targeting AAF in the CDC should be performed to confirm if these projections correspond to an other set of non auditory inputs to the auditory cortex of the deaf cat.

**References.**

1. Raczkowski D, Rosenquist AC (1983) Connections of the multiple visual cortical areas with the lateral posterior-pulvinar complex and adjacent thalamic nuclei in the cat. J Neurosci 3: 1912-1942.

2. Updyke BV (1981) Projections from visual areas of the middle suprasylvian sulcus onto the lateral posterior complex and adjacent thalamic nuclei in cat. J Comp Neurol 201: 477-506.

3. Lee CC, Winer JA (2008) Connections of cat auditory cortex: I. Thalamocortical system. J Comp Neurol 507: 1879-1900.

**Tables.**

| **Animal** | **Hemisphere** | **Tracer (µl)** | **Area** | **Observations** |
| --- | --- | --- | --- | --- |
| **CT10-NHC** | Right | FE simple (1.0) | DZ/ALLS | Excluded for quantification |
| **CT11-CDC** | Left | DY simple (0.3) | A1/AAF | Excluded for quantification |
| **CT16-CDC** | Left | DY simple (0.8) | AAF /A1 | Excluded for quantification |

Sup table 1 : Description of the supplementary injection sites.

| ***Case*** | | ***CT10 FE*** | | ***CT11 DY*** | | ***CT16 DY*** | |
| --- | --- | --- | --- | --- | --- | --- | --- |
| Number of Neurons  and Sections Used | | Nr | Sct | Nr | Sct | Nr | Sct |
| Thalamic nuclei | MGD | 74 | 9 | 78 | 4 | 273 | 6 |
|  | MGM | 108 | 12 | 321 | 12 | 650 | 18 |
|  | MGV | 223 | 13 | 1307 | 17 | 482 | 12 |
|  | Po | 76 | 6 | 4 | 1 | 452 | 8 |
|  | SG | 90 | 10 |  |  | 156 | 11 |
|  | VB |  |  | 1 | 1 | 5 | 9 |
|  | LP | 27 | 3 | 3 | 2 | 1 | 6 |
|  | Pul |  |  |  |  |  |  |
| **Thalamic labelling** |  | **598** | | **1714** | | **2019** | |

Sup. table 2 : Thalamic labeling.

| ***Case*** | | ***CT10 FE*** | | ***CT11 DY*** | | ***CT16 DY*** | |
| --- | --- | --- | --- | --- | --- | --- | --- |
| Number of Neurons  and Sections Used | | Nr | Sct | Nr | Sct | Nr | Sct |
|  | **Areas** |  |  |  |  |  |  |
| Tonotopic | A1 | 803 | 25 |  |  | 1640 | 11 |
|  | AAF | 114 | 10 |  |  |  |  |
|  | P | 28 | 7 | 62 | 3 | 159 | 4 |
|  | VP | 1 | 9 | 53 | 5 |  |  |
|  | Ve | 27 | 8 | 397 | 4 | 30 | 2 |
| ***Number of neurons*** | | ***973*** | | ***512*** | | ***1829*** | |
| Non Tonotopic | A2 | 65 | 14 | 543 | 9 | 1026 | 8 |
|  | AES | 415 | 14 | 431 | 10 | 419 | 3 |
|  | DZ |  |  | 734 | 13 | 1150 | 13 |
| ***Number of neurons*** | | ***351*** | | ***1708*** | | ***2595*** | |
| Limbic | Te | 0 | 5 | 86 | 4 | 15 | 2 |
|  | Insula | 99 | 19 | 29 | 6 | 12 | 6 |
|  | 35/36 | 0 | 5 | 123 | 14 |  |  |
| ***Number of neurons*** | | ***99*** | | ***81*** | | ***27*** | |
| Temporal | ED | 284 | 11 |  |  | 0 | 3 |
|  | EI | 128 | 12 | 35 | 3 | 25 | 1 |
|  | Ev | 4 | 10 | 46 | 3 | 30 | 4 |
| ***Number of neurons*** | | *416* | | ***81*** | | ***55*** | |
| Parietal SSS | ALLS |  |  | 263 | 12 | 117 | 11 |
|  | AMLS | 67 | 20 | 6 | 3 | 1 | 2 |
|  | PLLS | 100 | 11 |  |  | 3 | 3 |
|  | PMLS | 33 | 14 |  |  | 2 | 1 |
| ***Number of neurons*** | | ***200*** | | ***269*** | | ***123*** | |
| Posterior Ectosylvian Gyrus | 19 |  |  |  |  |  |  |
|  | 20 |  |  |  |  |  |  |
|  | 21 |  |  |  |  |  |  |
|  | PS |  |  |  |  |  |  |
| ***Number of neurons*** | | ***0*** | | ***0*** | | ***0*** | |
|  | |  | |  | |  | |
| Anterior AES | VAE |  |  |  |  |  |  |
|  | S-IV |  |  |  |  |  |  |
| ***Number of neurons*** | |  | | ***0*** | | ***0*** | |
| Somato-motor | 4 |  |  |  |  | 1 | 11 |
|  | 5 | 85 | 22 |  |  | 5 | 3 |
|  | 6 | 19 | 11 |  |  |  |  |
|  | 7 | 92 | 25 |  |  |  |  |
|  | S2 | 2 | 6 | 30 | 8 |  |  |
| ***Number of neurons*** | | ***198*** | | ***30*** | | ***6*** | |
| Others | PFC | 8 | 24 |  |  | 1 | 4 |
|  | CG | 46 | 30 |  |  | 2 | 1 |
| ***Number of neurons*** | | ***54*** | | ***0*** | | ***3*** | |
|  | |  | |  | |  | |
| ***Total Labelling*** | | ***2420*** | | ***2838*** | | ***4638*** | |

Sup. table 3 : Cortical labeling. Same convention as in Sup Table 2.

| ***Case*** | | ***CT10 FE*** | | ***CT11 DY*** | | ***CT16 DY*** | |
| --- | --- | --- | --- | --- | --- | --- | --- |
| Number of Neurons  and Sections Used | | Nr | Sct | Nr | Sct | Nr | Sct |
|  | **Areas** |  |  |  |  |  |  |
| Tonotopic | A1 | 5 | 3 | 148 | 9 | 37 | 9 |
|  | AAF |  | 10 | 39 | 5 | 319 | 6 |
|  | P |  | 7 | 25 | 1 |  |  |
|  | VP |  | 9 | 7 | 1 |  |  |
|  | Ve |  | 8 |  |  |  |  |
| Non Tonotopic | A2 | 5 | 1 |  |  |  |  |
|  | AES | 12 | 2 |  |  | 3 | 1 |
|  | DZ | 24 | 7 | 56 | 9 | 6 | 10 |
| Limbic | Te |  |  |  |  |  |  |
|  | Insula | 3 | 2 |  |  |  |  |
|  | 35/36 |  |  | 11 | 4 |  |  |
| Temporal | ED | 2 | 2 |  |  |  |  |
|  | EI |  |  |  |  |  |  |
|  | Ev |  |  |  |  |  |  |
| Parietal SSS | ALLS | 27 | 7 | 1 | 1 |  |  |
|  | AMLS | 2 | 1 |  |  |  |  |
|  | PLLS | 1 | 1 |  |  |  |  |
|  | PMLS |  |  |  |  | 2 | 2 |
| Posterior Ectosylvian Gyrus | 19 |  |  |  |  |  |  |
|  | 20 |  |  |  |  |  |  |
|  | 21 |  |  |  |  |  |  |
|  | PS |  |  |  |  |  |  |
| Anterior AES | VAE |  |  |  |  |  |  |
|  | S-IV |  |  |  |  |  |  |
| Somato-motor | 4 |  |  |  |  |  |  |
|  | 5 |  |  |  |  |  |  |
|  | 6 |  |  |  |  |  |  |
|  | 7 |  |  |  |  |  |  |
|  | S2 |  |  |  |  | 4 | 1 |
| Others | PFC |  |  |  |  |  |  |
|  | CG |  |  |  |  |  |  |
| ***Total Labelling*** | | **81** | | **287** | | **371** | |

Sup. table 4 : Cortical callosal labeling. Same convention as in Sup Table 3.
